# Comprehensive polar metabolomics and lipidomics profiling discriminates the transformed from the non-transformed state in colon tissue and cell lines

**DOI:** 10.1038/s41598-021-96252-4

**Published:** 2021-08-26

**Authors:** Caroline Rombouts, Margot De Spiegeleer, Lieven Van Meulebroek, Lynn Vanhaecke, Winnok H. De Vos

**Affiliations:** 1 Laboratory of Chemical Analysis, Department of Veterinary Public Health and Food Safety, Faculty of Veterinary Medicine, Ghent University, Salisburylaan 133, 9820 Merelbeke, Belgium; 2 Department of Molecular Biotechnology, Cell Systems and Imaging, Faculty of Bioscience Engineering, Ghent University, Coupure Links 653, 9000 Ghent, Belgium; 3 Laboratory of Cell Biology and Histology, Department of Veterinary Sciences, Faculty of Veterinary Medicine, Antwerp University, Universiteitsplein 1, 2610 Wilrijk, Belgium; 4 Institute for Global Food Security, School of Biological Sciences, Queen’s University, University Road, Belfast, BT7 1NN Northern Ireland, UK

**Keywords:** Liquid chromatography, Diagnostic markers, Cancer metabolism, Colorectal cancer, Metabolomics

## Abstract

Colorectal cancer (CRC) is the fourth most lethal disease worldwide. Despite an urgent need for therapeutic advance, selective target identification in a preclinical phase is hampered by molecular and metabolic variations between cellular models. To foster optimal model selection from a translational perspective, we performed untargeted ultra-high performance liquid chromatography coupled to high-resolution mass spectrometry-based polar metabolomics and lipidomics to non-transformed (CCD841-CON and FHC) and transformed (HCT116, HT29, Caco2, SW480 and SW948) colon cell lines as well as tissue samples from ten colorectal cancer patients. This unveiled metabolic signatures discriminating the transformed from the non-transformed state. Metabolites involved in glutaminolysis, tryptophan catabolism, pyrimidine, lipid and carnitine synthesis were elevated in transformed cells and cancerous tissue, whereas those involved in the glycerol-3-phosphate shuttle, urea cycle and redox reactions were lowered. The degree of glutaminolysis and lipid synthesis was specific to the colon cancer cell line at hand. Thus, our study exposed pathways that are specifically associated with the transformation state and revealed differences between colon cancer cell lines that should be considered when targeting cancer-associated pathways.

## Introduction

Colorectal cancer (CRC) is the second and third most diagnosed cancer in females and males, respectively, and the fourth leading cause of cancer-related mortality worldwide. The incidence rates are strongly variable throughout the world, whereby developed regions have more CRC patients than less developed countries. Disease risk is primarily associated with lifestyle factors, in particular Western habits such as the consumption of diets rich in fat and sugar, high in red and processed meat and limited physical activity^1,2^. Although the discovery of CRC-biomarkers and associated pathways has contributed to enhanced screening and improved treatment, the 5-year survival rate of metastatic CRC is still only 10%, emphasizing the need for better therapeutic strategies^3^.

In cancer research, animal models have been of great value for discovering novel biochemical pathways and drug testing. The most common models are xenograft and chemically (*e.g.,* azoxymethane) or genetically (*e.g., Apc* knockout) induced cancer rodent models^4–6^. In keeping with the 3R principles (Replacement, Reduction and Refinement), a framework that aims at minimizing animal use and suffering, researchers continuously seek *bona fide* alternatives wherever possible^7^. Therefore, many in vitro models, especially cell culture models, have been established to study CRC and therapeutic strategies^8^. Evidently, cell lines have less ethical constraints in comparison to animal models, and at the same time they are much easier in use, inexpensive and provide sufficient material, enabling rapid experimental progress^9^.

Whilst primary cell cultures from healthy patients may be considered to resemble the native state more closely, they are less practical to culture and have limited life span. Immortalized cell lines make a valuable alternative since they are still representative for the tissue of origin and are easier to handle and to maintain in culture^10^. For example, the CCD841-CON and Fetal Human Colon (FHC) cell lines are both immortalized, but non-transformed (NT) colon cell lines obtained from fetal normal colon mucosa^11–13^. On the other side of the spectrum, transformed (T) cell lines isolated from (adeno)carcinomas may serve as representative samples for studying different cancer stages. For example, HT29, SW480 and SW948 were established from colorectal adenocarcinomas, whilst Caco2 and HCT116 were established from colorectal carcinomas^14^. These cell lines have been extensively used to study regulatory mechanisms in CRC as well as for the purpose of identifying chemopreventive and -therapeutic agents^15^. Preclinical data using in vivo and in vitro models revealed that only 5% of candidate therapies demonstrate clinical efficacy in phase III trials. This high attrition rate can be attributed to some extent to the use of models that are not fully representative for the type of cancer of interest^9,16^. For example, it has been demonstrated that many cancer cell lines used to model various types of cancers were in fact derived from the HeLa cervical cancer cell line and not the corresponding tumor^17^. Moreover, it was also observed that long-term passaging of cells can lead to a reduced resemblance to the tumor of origin^13,14^. Therefore, several efforts have been made to reduce cell line misidentification, primarily by molecular phenotyping^15,18,19^. Although metabolites are the end-products in the gene-protein-metabolite cascade and thus key reporters of cellular activity, metabolic profiling or fingerprinting studies of cancer cells and tissue are scarce^20^. Pioneering studies have revealed a conspicuous metabolic rewiring in CRC. For example, both CRC tissue and cell lines have been found to display higher levels of phospholipids, of which certain (*e.g.,* phosphatidylcholine) correlate with metastatic propensity^21,22^. Also, specific nucleotides and carbohydrates have been found upregulated in CRC patient tissue^23^. The latter may represent metabolic evidence for a Warburg phenotype^21^, although higher rates of oxidative phosphorylation have been reported in CRC cell lines as well^23^. Thus, our understanding of the exact metabolic status of CRC cells is far from complete. And, to the best of our knowledge, no studies are available that assessed whether metabolic differences between healthy and cancerous colon tissue can be extrapolated to cell cultures and vice versa. We have previously optimized and validated an untargeted ultra-high performance liquid chromatography coupled to high-resolution mass spectrometry-based (UHPLC-HRMS) metabolomics and lipidomics method for human colon tissue and colon cell lines^24^. In this work, we exploited the approach to unveil pathways linked to the CRC transformation processes in vivo and in vitro and to provide novel insights into the metabolic phenotype of different cell lines that could foster the selection of the optimal one(s) towards specific targets in cancer drug research.

## Results

### Metabolic profiles differ between the NT and T state in colon cell lines and tissue

Untargeted UHPLC-HRMS metabolomics and lipidomics profiling was performed on samples of two NT (FHC and CCD 841 CON) and five T (HT29, Caco2, HCT116, SW480 and SW948) colon cell lines as well as on cancerous (T) and flanking non-cancerous (NT) tissue biopsy samples from 10 CRC patients. Of each cell line, cellular extracts were obtained from three culture flasks (technical replicates) at three different passages (P1-3), separated by one-week intervals (considered as biological replicates). To avoid metabolic shifts during storage from introducing bias between cell lines^25^, all cell lines of one passage were harvested at the same day, extracted and analyzed for metabolomics directly after. The remaining cell pellets were stored at − 80 °C and subjected for joint lipidomic extraction and analysis (all passages in one run). One sample of the FHC line (P3) was excluded for having too limited starting material. Chromatographic peak processing resulted in the detection of in total 879 metabolite and 17,432 lipid components, after removing those with a coefficient of variation (CV) > 30% in quality control (QC) samples composed of cell line or tissue matrix extracts. Valid orthogonal partial least squares discriminant analysis (OPLS-DA) models (Table 1) could be constructed for both colon cell line and tissue samples, allowing discrimination between the NT and T state^24^. For tissue, 196 and 722 discriminative components were retained for the NT and T state, respectively. For cell lines, 223 and 801 components were observed to be discriminative for the NT or T state, respectively. Of all 1942 retained components, 316 were discriminative for T or NT state in both matrices (tissue and cell lines), which implies they had a significant contribution to the resulting model (as indicated by a Variable Importance parameter (VIP) value > 1.0). Thus, the untargeted UHPLC-HRMS approach revealed a specific set of metabolomic components that typify the transformation state (NT vs T) of cells and tissue.

**Table 1:** Validation parameter values of OPLS-DA models using whole untargeted datasets (PC = Principal Components; IM = Ionization Mode).

Colon tissue samples	Nr PC	R^2^Y	Q^2^	CV-ANOVA *P* value	Permutation testing
Polar metabolomics (+ and – IM)	1 + 1 + 0	0.978	0.938	< 0.001	OK
Lipidomics (+ IM)	1 + 1 + 0	0.939	0.830	< 0.001	OK
Lipidomics (- IM)	1 + 2 + 0	0.897	0.713	< 0.001	OK

**Colon cell line samples**	**Nr of PC**	**R**^**2**^**Y**	**Q**^**2**^	**CV-ANOVA** **P value**	**Permutation testing**
Polar metabolomics (+ and – IM)	1 + 1 + 0	0.962	0.943	< 0.001	OK
Lipidomics (+ IM)	1 + 1 + 0	0.719	0.674	< 0.001	OK
Lipidomics (− IM)	1 + 1 + 0	0.962	0.943	0.0054	OK

### Downstream analysis uncovers metabolites specific to the NT and T state, but also reveals cell line-specific patterns

**Figure 1:**
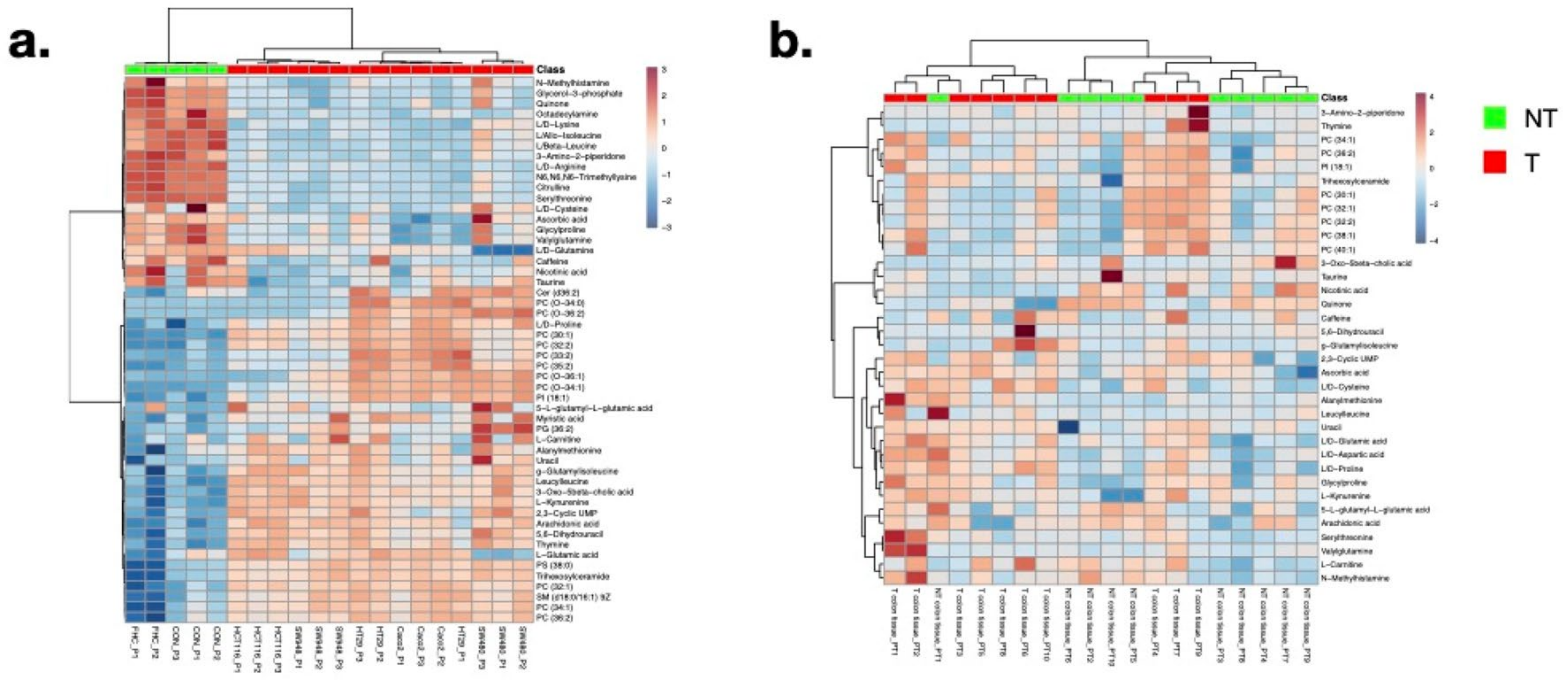
Heat map of metabolites that discriminate between the non-transformed and transformed state in (**a**) colon cell lines and (**b**) colon tissue. Normalization was performed by dividing the peak area (expressed by arbitrary units) of a specific component in a sample by the mean peak area of that component in the next two QC samples that were run after every ten samples. Hierarchical clustering was performed using Pearson correlation coefficient and Ward linkage. P1-3 = cell line passage 1–3 (1-week time interval, considered as replicate); PT1-10 = colorectal cancer patient 1–10.

The accurate mass of 1626 discriminative components was queried in the HMDB and LIPIDMAPS for putative identifiers (IDs). 377 IDs could be linked to components that were discriminative for the NT or T state in colon cell lines or tissue and were subsequently subjected to fragmentation experiments. This resulted in the identification of 55 metabolites with the highest levels of confidence*,* whereof 35 and 52 were discriminative (VIP value > 1.0 and/or *P* value < 0.05) for the transformation state in colon tissue and cell lines, respectively (Supplementary Tables S2-3). In total, 32 metabolites were shared between both datasets of which 17 and 3 were upregulated in the T or NT state, respectively. Twelve metabolites showed discrepancies between the two matrices (Supplementary Table S3). Hierarchical clustering of the identified metabolites by their normalized peak area revealed two main clusters of cell line samples, corresponding to NT and T state, and within the T state samples, three sub-clusters, one for Caco2 and HT29, one for HCT116 and SW948, and one for SW480 (Fig. 1a). The individual passages of all but one cell line (HT29) clustered together, emphasizing the reproducibility of the measurements. The discrimination between T and NT state was also visible for the tissue samples, albeit to a lesser extent (Fig. 1b). OPLS-DA models that were constructed using only the subset of discriminative metabolites (VIP value > 1.0 and/or *P *value < 0.05) for colon tissue and cell lines confirmed their potential to discriminate transformation states and differentiate HT29 and HCT116 from SW480 and HCT116 cell lines (Table 2). Thus, these observations demonstrate the relevance of the identified metabolites for classification of the different cell lines.

**Table 2:** Validation parameter values of OPLS-DA models using solely the discriminative metabolites. 35 and 52 discriminative metabolites (VIP value > 1.0 and/or *P* value > 0.05) were used to construct OPLS-DA models for colon tissue and cell lines, respectively.

Colon tissue samples	N° of principal components	R^2^Y	Q^2^	CV-ANOVA *P* value	Permutation testing
Non-transformed vs. transformed	1 + 1 + 0	0.837	0.597	< 0.01	OK

Colon cell line samples	N° of principal components	R^**2**^**Y**	Q^**2**^	CV-ANOVA *P* value	Permutation testing
Non-transformed vs. transformed	1 + 1 + 0	0.978	0.960	< 0.001	OK

Transformed colon cell line samples	N° of principal components	R^**2**^**Y**	Q^**2**^	CV-ANOVA *P* value	Permutation testing
Caco2 vs. HT29	1 + 1 + 0	0.996	0.707	0.405	NOK
Caco2 vs. HCT116	1 + 1 + 0	0.990	0.932	0.100	OK
Caco2 vs. SW480	1 + 1 + 0	0.987	0.929	0.105	OK
Caco2 vs. SW948	1 + 1 + 0	0.986	0.929	0.104	OK
HT29 vs. HCT116	1 + 1 + 0	1	0.980	0.030	OK
HT29 vs. SW480	1 + 1 + 0	0.999	0.909	0.134	OK
HT29 vs. SW948	1 + 1 + 0	0.997	0.954	0.068	OK
HCT116 vs. SW480	1 + 1 + 0	0.999	0.967	0.049	OK
HCT116 vs. SW948	1 + 1 + 0	0.995	0.934	0.097	OK
SW480 vs. SW948	1 + 1 + 0	0.989	0.959	0.061	OK

### Phospholipids are dominant differentiators within T cell lines

**Figure 2:**
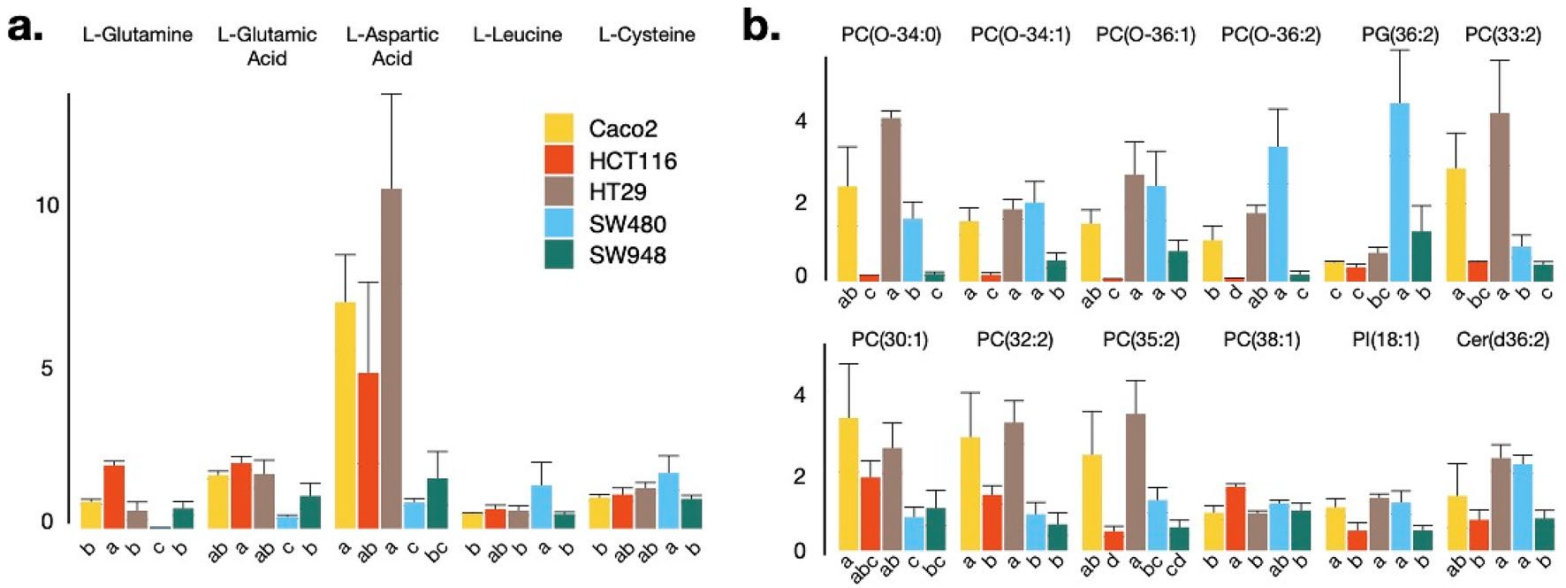
Significantly altered metabolites between different transformed cell lines: (**a**) polar metabolites and (**b**) lipids. Normalization was performed by dividing the peak area (expressed in arbitrary units) of a specific component in a sample by the mean peak area of that component in the next two QC samples that were run after every ten samples. Cell lines assigned with different letters are statistically (*P* value  < 0.05) different from each other. Error bars represent standard deviations (n = 3 replicates).

Clustering of the discriminative metabolites revealed a clear separation between different T cell lines (Fig. 1, Supplementary Table S2). To reveal the major contributors of this separation, metabolite levels were statistically compared between the different T cell line types. Of the 52 identified metabolites that were discriminative between the T and NT state, 15 were significantly (*P* value < 0.05) different between the cancer cell lines (Fig. 2). Of the polar metabolites, L/D-glutamine and L/D-glutamic acid and L-aspartic acid were particularly low, and L/β-leucine and L/D-cysteine proportionally high in the SW480 when compared to other cancer cell lines (Fig. 2a). Overall, lipids were much more abundant in the Caco2 and HT29 cell line as compared to the other cell lines, but in-between both cell lines no significant differences could be observed (Fig. 2b). The HCT116 and SW948 cell lines were especially low in lipids, except for PC (38:1), which was highly abundant in the HCT116 cell line. The SW480 cell line demonstrated the highest levels of PG (36:2), but PC (0–34:0), PC (O-34:1), PC (0–36:1), PC (O-36:2), PI (18:1) and Cer (d36:2) were detected at similar relatively high abundances in the Caco2 and HT29 cell lines, while PC (33:2), PC (30:1), PC (32:2) and PC (35:2) were detected at relatively low levels (Fig. 2b). Thus, phospholipid composition defines T cell line identity.

### Pathways associated with the NT or T state in colon tissue and cell lines

**Figure 3:**
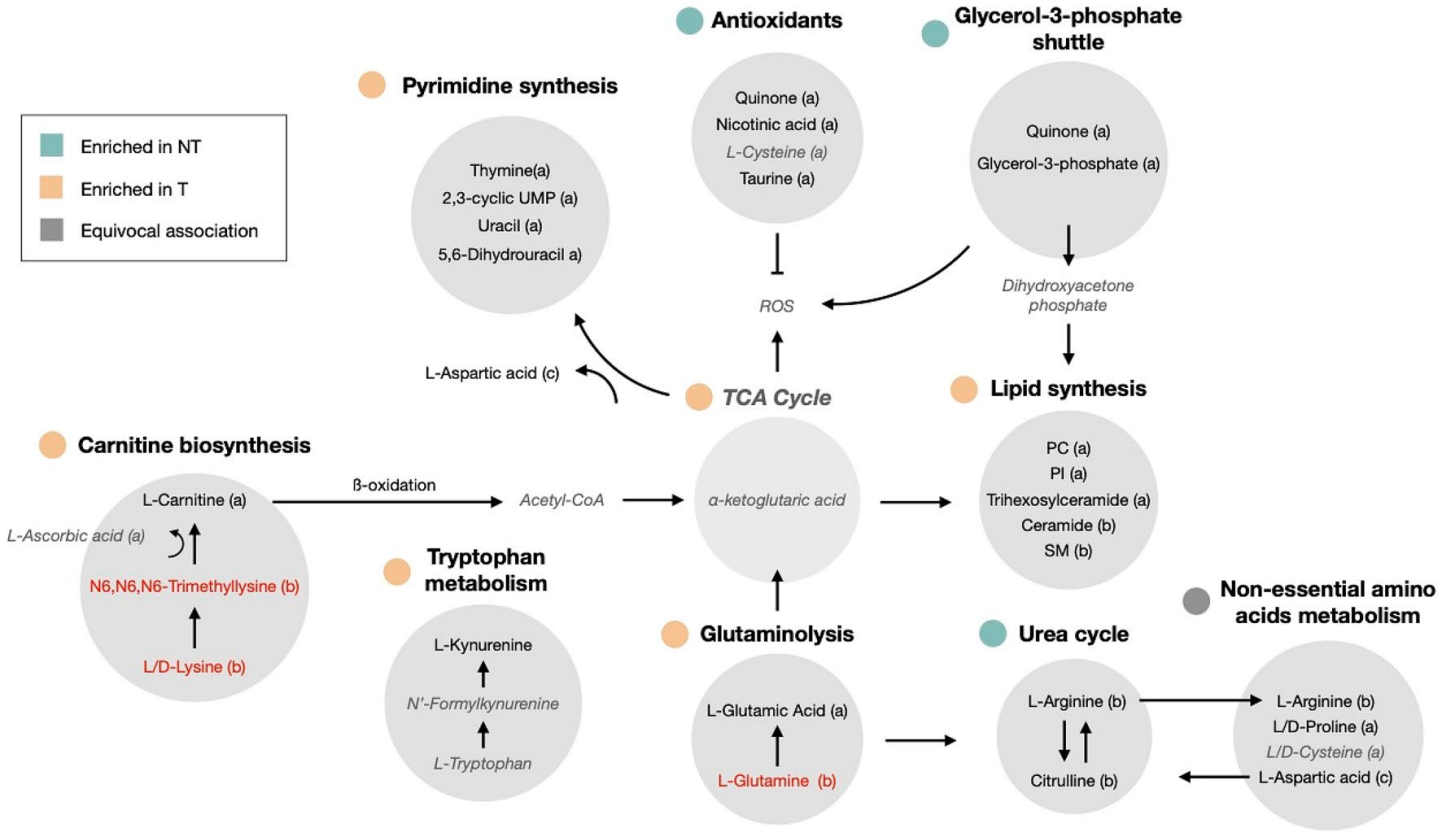
Schematic overview of the interactions between the identified metabolites and the involved pathways in non-transformed or transformed state. Metabolites that were discriminative for both matrices are indicated with (**a**), whereas those that were only found colon cell lines are indicated with (**b**) or tissue matrix with (**c**). Metabolites that are regulated in an opposite direction of the identified pathway are indicated in red and metabolites with conflicting changes between cell line and tissue samples or without a particular state association are written in lighter fray and italic.

To unveil the major pathways involved in carcinogenesis, quantitative enrichment analysis was performed for each matrix by introducing the QC-normalized abundances of the discriminative metabolites in MetaboAnalyst 4.0. This resulted in 49 and 33 significantly (FDR < 0.05) affected pathways for the colon cell line and tissue matrix, respectively (Supplementary Table S4-5). Thirty-two pathways were shared between both datasets as listed in Table 3. Remarkably, L-glutamic acid played a central role in 23 of these pathways (Table 3). To reveal metabolic networks and improve the understanding of the metabolic fluxes of the identified metabolites further a metabolic map was constructed, listing the most important metabolites and their direct interaction as well as their regulating role in transformation status considering their behavior in both cell lines and tissue (Fig. 3). Using this approach, glutaminolysis, pyrimidine synthesis, lipid synthesis, carnitine biosynthesis and tryptophan catabolism emerged as dominant processes in the T state, whereas urea cycle, glycerol-3-phosphate shuttle and antioxidant responses were more prominent in the NT state. Non-essential amino acids metabolism showed no clear association with a particular state (Fig. 3).

**Table 3:**
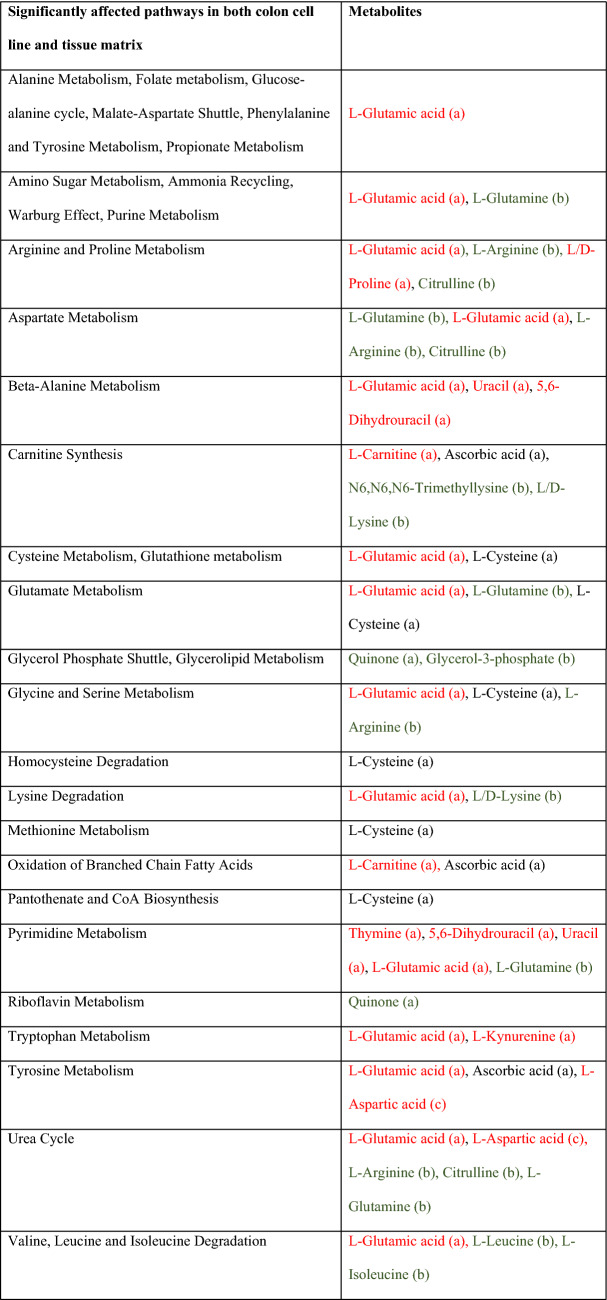
Quantitative enrichment analysis in MetaboAnalyst 4.0. Metabolites were upregulated in non-transformed (green) or transformed (red) state and discriminative for both matrices (a), colon cell line (b) or tissue matrix (c). Metabolites that behaved contradictory in colon cell line and tissue samples are presented in black.

### Extent of glutaminolysis varies between the different T cell line types

**Figure 4:**
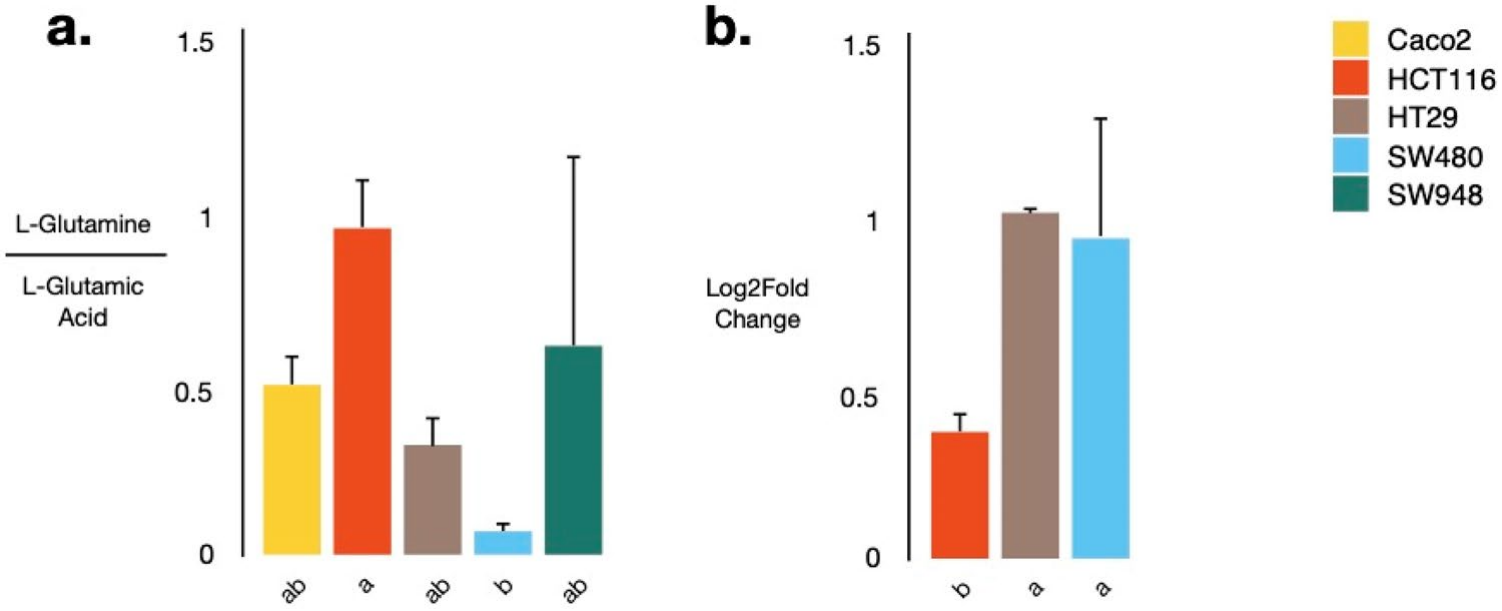
Conversion of L-glutamine to L-glutamic acid and expression of *GLS1 *in vitro. (**a**) Ratio L-glutamine/glutamic acid was determined by using the individual metabolite abundances in the transformed cell line samples; (**b**) Expression of *GLS1*, encoding glutaminase was assessed in the HCT116, HT29 and SW480 cell line by means of qPCR and expressed as log2 fold change relative to the HT29 cell line. Cell lines assigned with different letters are statistically (*P* value  < 0.05) different from each other. Error bars represent standard deviations (n = 3).

Based on the pathway analysis, we hypothesized that glutaminolysis plays a central role in the T state as opposed to the NT state in both matrices. Moreover, individual L-glutamine and L-glutamic acid levels were significantly different between the cancer cell lines. Therefore, as a case study, this pathway was investigated in more detail. First, the ratio of L-glutamine/L-glutamic acid was determined based on the detected metabolite levels in the samples (Fig. 4a) and was found to be significantly (*P* value  = 0.012) lower in the SW480 than in the HCT116 cell line samples, implying more conversion of L-glutamine to L-glutamic acid in the SW480 cell line. The HT29 and Caco2 cell lines demonstrated similar conversion rates, whilst the ratio was less consistent in the SW948 cell line, as indicated by the high standard deviation (Fig. 4a). To find out whether the higher conversion was due to an upregulation of glutaminase, the expression of the glutaminase-encoding gene *GLS1* was assessed by means of qPCR in the HCT116, SW480 and HT29 cell lines. This revealed that the expression of *GLS1* was significantly lower in the HCT116 as compared to the HT29 (*P* value  = 0.0031) and the SW480 cell line (*P* value  = 0.0056), aligning with the higher L-glutamine/L-glutamic acid ratio and the lower conversion of L-glutamine to L-glutamic acid in the HCT116 cell line (Fig. 4b).

## Discussion

In this work, untargeted metabolomics and lipidomics was performed on NT and T colon cell line and tissue samples to identify common molecular signatures. Based on the metabolic profiles, validated OPLS-DA models were constructed that allowed discrimination of the two states in both matrices and the identification of 35 and 52 discriminative metabolites in colon cell lines and tissue, respectively, of which 32 were shared between matrices. Glutaminolysis, pyrimidine synthesis, phospholipid synthesis, carnitine biosynthesis and tryptophan metabolism appeared to be hyperactivated in the T state, whereas urea cycle, glycerol-3-phosphate shuttle and antioxidant responses were more prominent in the NT state. The extent of glutaminolysis and lipid content were distinct between the different cell line types.

L-glutamic acid was characteristic for colon cancer cell lines and tumor tissue and is generated during glutaminolysis, *i.e.,* the degradation of L-glutamine. These results are in concordance with Hirayama et al. (2009), who observed higher L-glutamic acid and lower L-glutamine levels in colon tumor tissue, respectively^26^. Amongst the T cell lines, the SW480 cell line showed the highest rate of glutaminolysis as represented by higher conversion of L-glutamine towards L-glutamic acid. Also, qPCR results showed higher expression of glutaminase in this cell line indicating more enzyme availability promoting this metabolite conversion. Compared to HCT116 cells, SW480 cells displayed significantly lower L-glutamine and L-glutamic acid levels, which could indicate lower absorption of L-glutamine, but higher efficiency in utilizing this metabolite as an energy source for the latter. Interestingly, it has been shown that the removal of L-glutamine from media results in increased sensitivity of the SW480 as opposed to HCT116 and HT29 cell cultures, hence supporting the function of this metabolite as a more convenient energy source in the former^27^. L-leucine was more abundant in this cell line compared to the other cancer cell lines, which may hint towards increased activation of glutamate hydrogenase. Glutamate hydrogenase converts L-glutamic acid into α-ketoglutaric acid, an intermediate of the TCA cycle, thus promoting glutaminolytic activity^28^. Targeting glutaminolysis by inhibiting involved enzymes in cancer has gained interest and has already been proven to reduce the proliferation of cancer cells^29^.

Only recently, it has been shown that carbon and nitrogen of L-glutamine is incorporated into building blocks of cells, especially during high cell proliferation^30^. This is also in concordance with the higher presence of pyrimidines (*e.g.,* uracil and derivatives and thymine) and lipids (mainly PCs) in T samples^22,30,31^. In our study, especially saturated, mono-unsaturated and low-polyunsaturated PCs were discriminative for transformation, which aligns with a study that performed lipidomics on breast cancer and normal tissue and cell line samples^32^. Especially the HT29 and Caco2 cell line displayed relatively high PC levels. The HCT116 cell line was characterized by a lower abundance of lipid species, which may be linked to its decreased metastatic potential. Carcinogenesis has been linked with de novo lipogenesis and increased saturation of membrane lipids. The latter results in decreased membrane fluidity and permeability, which make cancer cells less prone to lipid peroxidation and chemotherapy in comparison to normal cells^22^. Based on this, it can be speculated that strategies to modulate membrane fluidity could improve the treatment of cancer^33^. Glycerol-3-phosphate, an intermediate in lipid synthesis, was detected at lower concentrations in the T state as opposed to the NT state, which further supports increased phospholipid biosynthesis in the T state^34^.

In our study, several antioxidants (taurine, nicotinic acid and quinone) were determined to be discriminative for NT samples in vitro and in vivo and are considered to be important in mitigating the damaging effects of ROS. These compounds act as a coenzyme in redox reactions, improve mitochondrial function resulting in less superoxide generation and scavenge superoxide radicals, respectively^35–37^. It is a common feature that ROS are elevated in cancer cells, where they are responsible for activating signaling pathways involved in cell proliferation^38^. However, when ROS are present in excessive amounts, they cause oxidative damage to macromolecules. To prevent this, cancer cells overproduce antioxidants^30^. Indeed, Kibi et al. (2019) observed higher taurine levels in CRC as opposed to adjacent tissue samples derived from 10 patients^39^. Therefore, one would intuitively expect that increased antioxidants would be linked to the T state, which was not the case in our work. Nevertheless, Hernandez-Lopez (2018) demonstrated that antioxidant responses between non-tumor adjacent tissue samples retrieved from patients with different stages of CRC differed, where non-tumor adjacent tissue of stage IV showed increased antioxidant levels in comparison to those of stage III^40^. In our work, 3 CRC patients had classification of tumors below stage II, whereas the tumors of the other 7 patients were classified in stage III and IV (Table 1). Hence, selection bias could have affected the antioxidant level interpretation.

The urea cycle is a metabolic pathway that is responsible for converting excess nitrogen from L-aspartic acid and ammonia, which results from amino acid catabolism, into urea. The urea cycle metabolites L-arginine and citrulline were downregulated in T cell lines as opposed to NT cell lines. These results are in accordance with Nagamani et al. (2016), who demonstrated that metabolic wiring accompanying carcinogenesis includes enhanced flux of L-aspartic acid towards pyrimidine synthesis and hence, decreased synthesis of L-arginine^41^.

Tryptophan metabolism involves the conversion of L-tryptophan into N’-formylkynurenine and L-kynurenine and this is depending primarily on indoleamine-2,3-oxygenase (IDO1), which is highly expressed in the colon. IDO-1 is one of the most upregulated in CRC and is strongly induced by inflammatory cytokines such as IFNγ^42^. L-kynurenine, which was elevated in the T state in colon tissue and cell lines, is associated with tumor immune escape and growth and hence, is considered as a hallmark of carcinogenesis^43^.

It has been reported that L-carnitine is elevated in cancer cells and associated with enhanced fatty acid uptake, oxidation and formation of ROS^38^. L-lysine is a precursor of N6,N6,N6-trimethyllysine, which can be converted to L-carnitine and further transformed to L-ascorbic acid. L-Carnitine is responsible for transporting fatty acids into the mitochondria or peroxisomes by conjugation with the acyl-CoA moiety to form acylcarnitines. Inside these cell organelles, acyl-CoA is released and enters the β-oxidation resulting in the formation of acetyl-CoA, that can in turn enter the TCA cycle^37^. In this study, only L-carnitine showed a clear association with the T state in both matrices. L-lysine, L-ascorbic acid and N6,N6,N6-trimethyllysine on the other hand were significantly elevated in the NT as opposed to the T state in cell line samples, but showed an opposite trend in the T colon tissue samples. Nevertheless, this observation was not significant for L-lysine and N6,N6,N6-trimethyllysine. Based on these findings, it can be concluded that L-carnitine biosynthesis and lysine degradation are higher in the T state. However, at the tissue level, such metabolic fluxes were less clearly observed, which can be due to genetic heterogeneity of samples. In concordance with these findings, elevated carnitine levels in plasma and tissue have been associated with breast, bladder and colon cancer, respectively^44–46^.

It is important to note that discrepancies were observed in metabolic profiles between the cell line and tissue samples. This may be due to the cell culture methodology that differs from the in vivo situation. For example, cell lines do not have a physiological extracellular matrix, 3D organization, and are much more homogeneous than tissue. Also, cell lines are adapted to their culture conditions, whereby media are composed in such a way that cell growth and viability are optimally supported^47^. Conversely, cells in tumor tissue are subjected to harsh conditions, including hypoxia and limited nutrient availability^47,48^. In this study, it was observed that L-ascorbic acid was significantly upregulated in the T as opposed to the NT tissue samples while a higher abundance was detected in NT as compared to the T cell line samples. In vivo trials have demonstrated that L-ascorbic acid is more abundant in cancer cells than normal cells through facilitated transport by glucose transporters, explaining the higher concentrations in colon cancer tissue. Nevertheless, this metabolite acts as a pro-oxidant in tumor cells, since these lack the enzyme catalase that converts L-ascorbic acid-induced ROS (H_2_O_2_) into water and oxygen^49^. Thus, cancer cells are more vulnerable than normal cells to the effects of this metabolite and can eventually stop growing or undergo apoptosis^50,51^. Hence, it could be hypothesized that cell lines have adapted to culture conditions by rewiring their utilized energy sources, for example through the absorption and use of L-glutamine, which is highly abundant in commercial cell culture media. This way, cancer cell lines are more protected against the detrimental effects of L-ascorbic acid. Indeed, recent research has shown that increased extracellular availability of glucose does not influence the rate of glucose utilization by cell line models. It was also demonstrated that higher glutamine concentrations in media resulted in higher glutamine fermentation in cell culture^52^.

In conclusion, despite the fact that in vitro cell culture models do not fully represent the in vivo situation, we discovered shared metabolites and corresponding pathways between colon tissue and cell lines involved in carcinogenesis. Glutaminolysis, associated pyrimidine and lipid synthesis, tryptophan catabolism, glycerol-3-phosphate shuttle and carnitine biosynthesis were enhanced in the malignant as opposed to the non-malignant state. In the NT state, urea cycle and antioxidant levels were more prominent. Moreover, it was clear that glutaminolysis, characterized by high expression of glutaminase and high conversion of L-glutamine to L-glutamic acid, was most abundant in the SW480 cell line as opposed to the other T cell lines. In addition, the HT29 and Caco2 cell lines contained the most saturated and one or double bonds-containing phospholipids, essential constituents of cell membranes and thus affecting membrane permeability towards cancer drugs. Hence, this work provides insights in pathways associated with colon carcinogenesis as well as valuable information to select the most appropriate cancer cell lines in the testing of novel chemotherapeutic agents.

## Materials and methods

### Biological samples

The human colorectal cell lines HT29, Caco2, HCT116, SW480 and SW948 (T state) and the immortalized colon cell lines FHC and CCD841-CON (NT state) were obtained from ATCC (Manassas, VA, U.S.A.). Dulbecco’s Modified Eagle’s Medium (DMEM), supplemented with 10% Fetal Bovine Serum (FBS) and 1% penicillin/streptomycin (P/S), was used to culture the cancer cell lines and CCD841-CON in a humidified incubator at 37 °C and 5% CO_2_/95% air. FHC cells were cultured in DMEM:F12 supplemented with 10 mM HEPES, 0.005 mg mL^-1^ insulin, 0.005 mg mL^-1^ transferrin, 100 ng mL^-1^ hydrocortison, 10% FBS and 1% P/S. All cell reagents were purchased from Life Technologies (Ghent, Belgium).

Cancerous colon material (100 mg) and corresponding healthy tissue (100 mg) from 10 individuals were provided by Biobank@UZA (Antwerp, Belgium; ID: BE71030031000; Belgian Virtual Tumorbank funded by the National Cancer Plan). The use of human colon tissue samples in this study was ethically approved by the University Hospital of Antwerp (ECD 16/37/368, Antwerp, Belgium) and patient details are listed in Supplementary Table S1.

### Metabolomics and lipidomics fingerprinting

In recent work, the applied two-step metabolomics and lipidomics extraction protocols for colon (cancer) cell lines and tissue were successfully optimized and validated. More information about the optimized parameters and validation experiments can be consulted in Rombouts et al*.* (2019)^24^. For each matrix (*i.e.,* cell lines and tissue samples), quality control (QC) samples were made from a pool of the matrix-specific samples and run in duplicate after every 10 samples.

Analytical standards were purchased from Sigma-Aldrich (St-Louis, Missouri, USA), ICN Biomedicals Inc. (Solon, Ohio, USA), TLC PharmChem (Vaughan, Ontario, Canada) or Cambridge Isotope Laboratories Inc. (Tewksbury, Massachusetts, USA). Detailed information about chromatographic and mass spectrometric features of the reference metabolite and lipid compounds can be consulted in De Paepe et al. (2018) and Van Meulebroek et al. (2017), respectively^53,54^. Solvents were of LC–MS grade for extraction purposes and obtained from Fisher Scientific (Loughborough, UK) and VWR International (Merck, Darmstadt, Germany). Ultrapure water was obtained by usage of a purified-water system (VWR International, Merck, Darmstadt, Germany).

An Ultimate 3000 XRS UHPLC system (Thermo Fisher Scientific, San José, CA, USA) and a Q-Exactive™ stand-alone bench top Orbitrap mass spectrometer (MS) (Thermo Fisher Scientific, San José, CA, USA), equipped with a heated electrospray ionization source (HESI II) operating in polarity switching mode, were used for polar metabolomics and lipidomics analysis as described by De Paepe et al. (2018) and Van Meulebroek et al. (2017), respectively^52,53^. For fragmentation experiments, the following MS/MS settings were applied during parallel reaction monitoring (PRM): resolution of 17,500 Full Width at Half maximum (FWHM), Automatic Gain Control (AGC) target of 2 × e^4^ ions, maximum injection time of 40 ms and an isolation window of 2.0 m/*z*.

### Data analysis

All cellular LC–MS data, together with tissue LC–MS data were processed simultaneously using Compound Discoverer™ 2.1 or Sieve™ 2.2 software (both obtained from Thermo Fisher Scientific, San Jose, CA, USA) for the metabolomics and lipidomics experiment, respectively. Herein, chromatographic peak alignment and component extraction was performed as described earlier^24^. In brief, components were identified using the following criteria: a minimum peak intensity of 500,000 au, minimum signal-to-noise ratio of 10, minimum number of isotopes of 2, retention time window of 0.75 min, maximum retention time shift of 0.25 min and maximum mass shift at 5 ppm. This resulted in the construction of one metabolomics dataset (two ionization modes combined) and two lipidomics datasets (positive and negative ionization modes separately). Next, unsupervised hierarchical clustering (Pearson correlation coefficient, Ward linkage) and multivariate statistical analysis (SIMCA™ 14.1 software, Umetrics AB, Umea, Sweden) were performed, whereby OPLS-DA models were built to discriminate the NT from the T state in tissue and cell lines separately. For modeling, data were log-transformed and Pareto-scaling was applied^55^. Validity of the obtained OPLS-DA models was evaluated based on several parameters including R^2^Y (> 0.5), Q^2^ (> 0.5), CV-ANOVA (p < 0.05), as well as permutation testing (n = 100)^56^. Selection of components that discriminated between the NT and T state in cell lines and tissue was based on a Variable Importance in Projection (VIP) score > 1.0 and a Jack Knife confidence interval that did not include 0^57^. Screening of components of interest was established based on accurate mass (Δ ppm < 6 ppm) in the Human Metabolome Database (HMDB) and LIPIDMAPS^58,59^. For those components that could be linked to putative IDs, fragmentation experiments were conducted, and MS/MS profiles were scored in CSI FingerID or compared with these of in-house analytical standards^60^. Components were then classified into different levels of assignment according to the recommendations for standard metabolite identification from the Chemical Analysis Working Group^61^. Based on this, retained compounds were described as identified compound (type 1; based on accurate mass, RT and MS/MS spectra that matched these of an analytical standard) or putatively annotated compound (type 2; based on accurate mass and MS/MS spectra that matched these of in silico MS/MS data generated with CSI FingerID).

### Statistical evaluation

Identified and putatively annotated metabolites were statistically evaluated (*P* value  < 0.05) in MetaboAnalyst 4.0, thereby using the independent two-sample or paired sample Wilcoxon test for, data from the colon cell lines and tissue samples, respectively. To this end, metabolites obtained from the tissue matrix were also statistically evaluated in the cell line matrix and vice versa. This approach allowed pinpointing metabolites relevant for discrimination between NT and T state in tissue or cell lines as well as those relevant in both matrices making extrapolation and comparison easier. Significantly different metabolites between the transformed cell line types were assessed using One-Way Anova and Tukey-adjusted post hoc testing for all pairwise comparisons, whereby *P* value s < 0.05 were considered significant.

### Data visualization, enrichment and pathway analysis

Heat maps presenting the abundances of the discriminative metabolites (VI* P* value  > 1.0 and/or *P* value  < 0.05) were constructed and hierarchical clustering (Pearson correlation coefficient and Ward linkage) was performed of the colon cell line and tissue samples in MetaboAnalyst 4.0 (www.metaboanalyst.ca). This software program was also used for quantitative enrichment analysis using biologically relevant human metabolic pathways of the Small Molecular Pathway Database (SMDB)^62^. Pathways with a false discovery rate (FRD) < 0.05 were considered significantly altered. Next to this, the KEGG Compound Database (www.genome.jp/kegg/compound) and scientific literature was consulted to provide a broader overview of transformation-associated pathways in colon tissue and cell lines.

### Quantitative PCR

RNA was extracted using the Nucleospin Triprep kit (Macherey–Nagel, Düren, Germany) and quality control was performed with the NanoDrop 2000 (Thermo Fisher Scientific, San Jose, CA, USA). Next, 1 μg of RNA was converted to cDNA synthesis with Superscript® II Reverse Transcriptase (RT) (Life Technologies, 18064-014) (Thermo Fisher Scientific, San Jose, CA, USA). qPCR reactions were performed on a CFX Connect™ system (Bio-Rad, California, USA) using SsoAdvanced™ Universal SYBR^®^ Green Supermix (Bio-Rad, California USA) according to the manufacturer’s instructions. Primers were used for *GLS1* (forward: CTCCAAGAATACCAAGTC; reverse: TTACAACAATCCATCAAGA) and reference genes *GADPH* (forward: GAAGGTGAAGGTCGGAGTCAAC; reverse: CAGAGTTAAAAGCAGCCCTGGT), *ACTB* (forward:CCTTGCACATGCCGGAG; reverse: GCACAGAGCCTCGCCTT) and *TUB* (forward: AGCAAGAGGGCGATTCCCTT; reverse: GGGAAGACACGCCCTGAAAG)^63^. qPCR technical replicates (n = 4) that deviated more than 0.3 Cq units and genes with inconsistent melting curves were removed from downstream analysis. Relative quantification was performed using the 2^−ΔΔCT^ method, whereby the HT29 samples were used as reference^64^. One-way ANOVA together with Tukey-adjusted post hoc testing for all pairwise comparisons was performed in RStudio 2.1, whereby *P* value s < 0.05 were considered significant.

## Supplementary Information

Supplementary Information.
