# Supplementary material for: Comprehensive polar metabolomics and lipidomics profiling discriminates the transformed from the non-transformed state in colon tissue and cell lines
Source: Sci Rep. 2021 Aug 26;11:17249. doi: 10.1038/s41598-021-96252-4 (PMC8390467; doi:10.1038/s41598-021-96252-4)
Supplement: Supplementary file 1 — Supplementary Information. [file 41598_2021_96252_MOESM1_ESM.pdf]

Supplementary Data

Comprehensive polar metabolomics and lipidomics profiling discriminates the transformed from the non-transformed state in colon tissue and cell lines

Caroline Rombouts [a,b,c], Margot De Spiegeleer [a], Lieven Van Meulebroek [a], Lynn Vanhaecke [a,d *], Winnok H. De Vos [c *]

[a] Ghent University, Faculty of Veterinary Medicine, Department of Veterinary Public Health and Food Safety, Laboratory of Chemical Analysis, Salisburylaan 133, B-9820 Merelbeke, Belgium

[b] Ghent University, Faculty of Bioscience Engineering, Department of Molecular Biotechnology, Cell Systems & Imaging, Coupure Links 653, 9000 Ghent, Belgium

[c] Antwerp University, Faculty of Veterinary Medicine, Department of Veterinary Sciences, Laboratory of Cell Biology & Histology, Universiteitsplein 1, 2610 Wilrijk, Belgium

[d] Institute for Global Food Security, School of Biological Sciences, Queen’s University, University Road, Belfast BT7 1NN, Northern Ireland, United Kingdom

* Shared senior authorship

Supplementary Tables

Supplementary Table S1. Information about disease stage of colorectal cancer patients.

**Patient**	**Colorectal cancer stage**	**TNM**
1	IIIC	T3N2bMx
2	IIIB	T3N2aMx
3	I	T2N0Mx
4	IIIC	T3N1aMx
5	IIIC	T3N2bMx
6	I	T2N0
7	IVA	T2N0M1
8	IIIB	T3N1
9	IIIB	T4N1bMx
10	IIA	T3N0Mx

TNM: Tumor Node Metastatis

Supplementary Table S2. Identity of compounds discriminative between the transformed and non-transformed state in colon tissue and cell lines. Type 1: Identified compound based on accurate mass, RT (retention time) and MS/MS spectra that matched these of an analytical standard; Type 2: Putatively identified compound based on accurate mass and MS/MS spectra that matched these of CSI FingerID). *Amino acids were identified based on corresponding L-isomers present in authentical commercial standards, but other isomeric forms cannot be fully excluded. PI = phosphatidylinositol, PC = phosphatidylcholine, SM = sphingomyeline, Cer = ceramide, PS = phosphatidylserine, PG = phosphatidylglycerol.

**Compound name**	**Detection method**	**Chemical formula**	**Main ionization adduct**	***m/z***	**RT (min)**	**Δ ppm**	**Level of identification (% similarity with predicted fragmentation spectrum in CSI:FingerID)**
Quinone	Metabolomics	$C_6H_4O_2$	$[M-H]^-$	107.01413	1.04	2.58	Type 1
Nicotinic acid	Lipidomics	$C_6H_5NO_2$	$[M+H]^+$	124.03955	1.27	1.98	Type 1
Taurine	Lipidomics	$C_2H_7NO_3S$	$[M+H]^+$	126.02215	1.33	1.67	Type 1
Arachidonic acid	Metabolomics	$C_{20}H_{32}O_2$	$[M-H]^-$	303.23281	14.06	0.48	Type 1
3-Oxo-5beta-cholic acid	Metabolomics	$C_{24}H_{38}O_3$	$[M+H]^+$	375.28892	11.68	1.20	Type 2 (71.49 %)
5-L-glutamyl-L-glutamic acid	Metabolomics	$C_{10}H_{16}N_2O_7$	$[M+H]^+$	277.10283	1.19	0.71	Type 2 (91.16 %)
Leucylleucine	Metabolomics	$C_{12}H_{24}N_2O_3$	$[M+H]^+$	245.18579	6.73	0.73	Type 2 (88.13 %)
3-Amino-2-piperidone	Metabolomics	$C_5H_{10}N_2O$	$[M+H]^+$	115.08689	0.92	2.62	Type 1
Ascorbic acid	Metabolomics	$C_6H_8O_6$	$[M-H]^-$	175.02394	1.26	4.98	Type 1
Glycylproline	Metabolomics	$C_7H_{12}N_2O_3$	$[M+H]^+$	173.09206	1.31	0.05	Type 2 (82.32 %)
L/D-Cysteine*	Metabolomics	$C_3H_7NO_2S$	$[M+H]^+$	122.02703	1.02	0.04	Type 1
N-methylhistamine	Metabolomics	$C_6H_{11}N_3$	$[M+H]^+$	126.10284	1.07	2.11	Type 2 (84.68 %)
Serylthreonine	Metabolomics	$C_7H_{14}N_2O_5$	$[M+H]^+$	207.09683	0.96	3.46	Type 2 (78.32 %)
Valylglutamine	Metabolomics	$C_{10}H_{19}N_3O_4$	$[M+H]^+$	246.14451	1.30	1.31	Type 2 (73.96 %)
Caffeine	Lipidomics	$C_8H_{10}N_4O_2$	$[M+H]^+$	195.08769	1.66	0.20	Type 1
2,3-Cyclic UMP	Metabolomics	$C_9H_{11}N_2O_8P$	$[M+H]^+$	307.03249	1.41	0.28	Type 2 (78.75 %)
5,6-Dihydrouracil	Metabolomics	$C_4H_6N_2O_2$	$[M+H]^+$	115.05046	1.9	2.23	Type 1
Uracil	Metabolomics	$C_4H_4N_2O_2$	$[M+H]^+$	113.03490	1.42	3.07	Type 1
Thymine	Metabolomics	$C_5H_6N_2O_2$	$[M+H]^+$	127.05025	2.57	0.37	Type 1

Alanylmethionine	Metabolomics	$C_8H_{16}N_2O_3S$	$[M+H]^+$	221.09533	2.47	0.49	Type 2 (71.89 %)
g-Glutamylisoleucine	Metabolomics	$C_{11}H_{20}N_2O_5$	$[M+H]^+$	261.14412	6.08	1.45	Type 2 (92.64 %)
L-Kynurenine	Metabolomics	$C_{10}H_{12}N_2O_3$	$[M+H]^+$	209.09194	4.09	0.61	Type 1
L/D-Proline*	Metabolomics	$C_5H_9NO_2$	$[M+H]^+$	116.07086	1.06	2.20	Type 1
L/D-Glutamic acid*	Metabolomics	$C_5H_9NO_4$	$[M+H]^+$	148.06043	0.98	0.03	Type 1
L-Carnitine	Metabolomics	$C_7H_{15}NO_3$	$[M+H]^+$	162.112367	0.99	0.63	Type 1
PI (18:1)	Lipidomics	$C_{27}H_{51}O_{12}P$	$[M-H]^-$	597.305	4.54	0.77	Type 2 (84.44 %)
PC (34:1)	Lipidomics	$C_{42}H_{82}NO_8P$	$[M+H]^+$	760.584486	9.00	0.78	Type 2 (92.97%)
PC (32:1)	Lipidomics	$C_{40}H_{78}NO_8P$	$[M+H]^+$	732.553076	8.55	0.96	Type 2 (92.33%)
PC (32:2)	Lipidomics	$C_{40}H_{76}NO_8P$	$[M+H]^+$	730.537456	8.32	0.92	Type 2 (91.14%)
PC (30:1)	Lipidomics	$C_{38}H_{74}NO_8P$	$[M+H]^+$	704.521816	8.08	0.94	Type 2 (88.11%)
PC (36:2)	Lipidomics	$C_{44}H_{84}NO_8P$	$[M+H]^+$	786.600166	9.23	0.72	Type 2 (91.33%)
Trihexosylceramide (d18:1/24:0)	Lipidomics	$C_{60}H_{113}NO_{18}$	$[M+H]^+$	1136.80572	9.54	2.36	Type 2 (72.03%)
L/D-Glutamine*	Metabolomics	$C_5H_{10}N_2O_3$	$[M+H]^+$	147.076406	0.95	0.08	Type 1
L/D-Arginine*	Metabolomics	$C_6H_{14}N_4O_2$	$[M+H]^+$	175.11890	0.94	0.30	Type 1
L/D-Lysine*	Metabolomics	$C_6H_{14}N_2O_2$	$[M+H]^+$	147.11277	0.86	0.23	Type 1
Citrulline	Metabolomics	$C_6H_{13}N_3O_3$	$[M+H]^+$	176.10267	0.98	1.69	Type 2 (86.59 %)
N6,N6,N6-Trimethyl-L-lysine	Metabolomics	$C_9H_{20}N_2O_2$	$[M+H]^+$	189.15978	0.94	0.14	Type 2 (71.20 %)
Glycerol-3-phosphate	Metabolomics	$C_3H_9O_6P$	$[M+H]^+$	173.02105	0.98	0.57	Type 1
L/ β -Leucine*	Metabolomics	$C_6H_{13}NO_2$	$[M+H]^+$	132.10199	2.16	0.65	Type 1
L/Allo-Isoleucine*	Metabolomics	$C_6H_{13}NO_2$	$[M+H]^+$	132.10198	2.37	0.57	Type 1
Octadecylamine	Lipidomics	$C_{18}H_{39}N$	$[M+H]^+$	270.31529	8.21	0.87	Type 2 (74.70 %)
SM (d34:1)	Lipidomics	$C_{39}H_{79}N_2O_6P$	$[M+H]^+$	703.57434	7.94	0.73	Type 2 (88.09 %)
Myristic acid	Lipidomics	$C_{14}H_{28}O_2$	$[M-H]^-$	227.20131	8.98	1.51	Type 2 (71.18 %)
Cer (d36:2)	Lipidomics	$C_{42}H_{81}NO_3$	$[M-H]^-$	646.6143	9.6	0.11	Type 2 (89.10 %)
PC (O-34:0)	Lipidomics	$C_{42}H_{86}NO_7P$	$[M+H]^+$	748.620736	9.42	0.98	Type 2 (87.73%)
PC (33:2)	Lipidomics	$C_{41}H_{78}NO_8P$	$[M+H]^+$	744.553026	8.552	1.01	Type 2 (91.15%)
PC (35:2)	Lipidomics	$C_{43}H_{82}NO_8P$	$[M+H]^+$	772.584226	9.016	1.11	Type 2 (90.62%)
PC (O-36:2)	Lipidomics	$C_{44}H_{86}NO_7P$	$[M+H]^+$	772.620806	9.418	0.86	Type 2 (71.61%)
PC (O-34:1)	Lipidomics	$C_{42}H_{84}NO_7P$	$[M+H]^+$	746.605026	9.194	1.06	Type 2 (93.30%)
PC (O-36:1)	Lipidomics	$C_{44}H_{88}NO_7P$	$[M+H]^+$	774.636426	9.666	0.89	Type 2 (86.86%)
PS (38:0)	Lipidomics	$C_{44}H_{86}NO_{10}P$	$[M-H]^-$	818.592394	8.974	0.90	Type 2 (79.07%)
PG (36:2)	Lipidomics	$C_{42}H_{79}O_{10}P$	$[M-H]^-$	773.534424	7.45	0.79	Type 2 (96.01%)
L/D-Aspartic acid*	Metabolomics	$C_4H_7NO_4$	$[M+H]^+$	134.04481	0.94	0.17	Type 1

PC (38:1)	Lipidomics	$C_{46}H_{90}NO_8P$	$[M+H]^+$	816.647206	9.9	0.58	Type 2 (90.03%)
PC (40:1)	Lipidomics	$C_{48}H_{94}NO_8P$	$[M+H]^+$	844.678396	10.41	0.69	Type 2 (88.93%)

Supplementary Table S3. Statistical values of compounds discriminative between the transformed and non-transformed state. *Amino acids were identified based on corresponding L-isomers present in authentical commercial standards, nevertheless other isomeric forms cannot be fully excluded. NT = non-transformed, T = transformed, PI = phosphatidylinositol, PC = phosphatidylcholine, SM = sphingomyeline, Cer = ceramide, PS = phosphatidylserine, PG = phosphatidylglycerol. NS: Not significant.

**Compound name**	**Ratio (NT/T colon cell lines)**	**P-value NT vs T colon cell lines**	**VIP-value colon cell line matrix**	**Ratio (NT/T colon tissue)**	**P-value NT vs T colon tissue**	**VIP-value colon tissue matrix**
Quinone	1.85	9.03E-04	1.17	2.64	5.86E-03	1.62
Nicotinic acid	2.06	2.54E-02	1.06	1.76	NS	1.75
Taurine	1.68	1.94E-02	0.94	3.05	NS	1.59
Arachidonic acid	0.69	1.29E-04	0.80	1.86	NS	1.23
3-Oxo-5beta-cholic acid	0.71	1.29E-04	0.78	3.04	4.88E-02	1.96
5-L-glutamyl-L-glutamic acid	0.90	2.54E-02	0.50	1.79	NS	1.18
Leucylleucine	0.71	1.29E-04	0.78	1.44	NS	1.56
3-Amino-2-piperidone	2.35	1.29E-04	1.28	0.67	1.95E-03	1.25
Ascorbic acid	1.07	1.07E-02	0.30	0.45	3.91E-03	1.51
Glycylproline	1.23	1.55E-03	0.53	0.46	1.95E-02	1.43
L/D-Cysteine*	1.83	1.94E-02	0.83	0.47	3.71E-02	1.36
N-methylhistamine	1.39	1.55E-03	0.90	0.79	4.88E-02	1.03
Serylthreonine	2.33	1.29E-04	1.38	0.65	3.91E-03	1.19
Valylglutamine	1.23	1.55E-03	0.50	0.55	NS	1.41
Caffeine	1.71	5.42E-03	1.17	0.40	NS	2.04
2,3-Cyclic UMP	0.86	1.29E-04	0.43	0.30	2.73E-02	1.70
5,6-Dihydrouracil	0.70	1.29E-04	0.80	0.19	NS	2.66
Uracil	0.81	1.29E-04	0.51	0.50	1.95E-03	1.70
Thymine	0.70	1.29E-04	0.81	0.19	NS	3.90
Alanylmethionine	0.86	2.45E-03	0.45	0.59	2.73E-02	1.40
g-Glutamylisoleucine	0.77	1.29E-04	0.65	0.21	3.71E-02	1.66
L-Kynurenine	0.71	1.29E-04	0.79	0.44	3.71E-02	1.48
L/D-Proline*	0.21	1.29E-04	1.39	0.64	9.77E-03	1.24
L/D-Glutamic acid*	0.16	3.28E-02	0.75	0.73	5.86E-03	0.92

L-Carnitine	0.63	3.28E-02	0.49	0.75	1.95E-03	0.95
PI (18:1)	0.15	1.29E-04	2.85	0.46	1.95E-02	1.27
PC (34:1)	0.19	1.29E-04	1.69	0.74	4.88E-02	0.87
PC (32:1)	0.09	1.29E-04	1.85	0.62	NS	1.34
PC (32:2)	0.06	2.58E-04	2.04	0.50	NS	1.81
PC (30:1)	0.02	2.58E-04	2.07	0.50	NS	1.92
PC (36:2)	0.17	1.29E-04	1.82	0.69	4.88E-02	1.14
Trihexosylceramide (d18:1/24:0)	0.10	1.29E-04	1.84	0.66	NS	1.17
L/D-Glutamine*	2.84	3.74E-03	1.37	1.28	NS	0.74
L/D-Arginine*	14.37	1.29E-04	2.53	1.04	NS	0.16
L/D-Lysine*	2.70	1.29E-04	1.42	0.98	NS	0.25
Citrulline	2.14	1.29E-04	1.33	0.81	NS	0.89
N6,N6,N6-Trimethyl-L-lysine	2.39	1.29E-04	1.36	0.82	NS	0.71
Glycerol-3-phosphate	1.94	1.29E-04	1.24	1.09	NS	0.93
L/β-Leucine*	10.29	1.29E-04	2.41	1.02	NS	0.44
L/Allo-Isoleucine*	7.54	1.29E-04	2.23	0.85	NS	0.26
Octadecylamine	5.99	1.29E-04	2.27	0.80	NS	0.85
SM (d34:1)	0.12	1.29E-04	1.95	0.78	NS	0.89
Myristic acid	0.58	1.29E-04	1.90	0.92	NS	0.32
Cer (d36:2)	0.53	3.74E-03	1.90	0.78	NS	0.40
PC (O-34:0)	0.09	1.46E-02	1.86	1.07	NS	0.17
PC (33:2)	0.10	3.74E-03	1.91	0.87	NS	0.87
PC (35:2)	0.07	5.16E-04	2.02	0.91	NS	0.41
PC (O-36:2)	0.06	1.07E-02	1.87	1.23	NS	0.62
PC (O-34:1)	0.03	9.03E-04	2.04	0.97	NS	0.08
PC (O-36:1)	0.04	3.74E-03	2.13	1.33	NS	0.62
PS (38:0)	0.10	1.29E-04	3.17	0.83	NS	0.55
PG (36:2)	0.14	1.29E-04	2.73	0.87	NS	0.32
L/D-Aspartic acid*	0.26	NS	0.91	0.70	1.13E-02	0.86
PC (38:1)	0.89	NS	0.07	0.46	NS	1.73
PC (40:1)	1.20	NS	0.73	0.45	NS	2.13

Supplementary Table S4. Quantitative enrichment analysis in MetaboAnalyst 4.0 (colon cell line matrix). 52 identified metabolites discriminating (VIP-value > 1.0 and/or P-value < 0.05) between the non-transformed and the transformed state in the colon cell line matrix were used for quantitative enrichment analysis. FDR = false discovery rate.

**Affected pathways in the colon cell line matrix**	**Total compounds in pathway**	**Hits**	**FDR**
Carnitine Synthesis	22	4	7.48E-10
Arginine and Proline Metabolism	53	4	7.48E-10
Urea Cycle	29	4	5.40E-09
Aspartate Metabolism	35	4	5.40E-09
Glycine and Serine Metabolism	59	3	1.83E-08
Alpha Linolenic Acid and Linoleic Acid Metabolism	19	1	2.83E-07
Biotin Metabolism	8	1	4.79E-07
Valine, Leucine and Isoleucine Degradation	60	3	6.13E-07
Phospholipid Biosynthesis	29	1	6.52E-07
Mitochondrial Electron Transport Chain	19	1	6.52E-07
De Novo Triacylglycerol Biosynthesis	9	1	6.52E-07
Cardiolipin Biosynthesis	11	1	6.52E-07
Glycerolipid Metabolism	25	2	2.43E-06
Glycerol Phosphate Shuttle	11	2	2.43E-06
Riboflavin Metabolism	20	1	1.32E-05
Fatty Acid Biosynthesis	35	1	3.57E-04
Lysine Degradation	30	2	5.72E-04
Pyrimidine Metabolism	59	5	7.24E-04
Caffeine Metabolism	24	1	2.83E-03
Beta-Alanine Metabolism	34	3	3.36E-03
Nicotinate and Nicotinamide Metabolism	37	3	7.60E-03
Arachidonic Acid Metabolism	69	2	7.89E-03
Tryptophan Metabolism	60	2	8.85E-03
Taurine and Hypotaurine Metabolism	12	2	9.43E-03
Glutamate Metabolism	49	3	1.09E-02
Cysteine Metabolism	26	2	1.59E-02

Glutathione Metabolism	21	2	1.59E-02
Histidine Metabolism	43	2	1.86E-02
Bile Acid Biosynthesis	65	1	1.86E-02
Pantothenate and CoA Biosynthesis	21	1	1.86E-02
Methionine Metabolism	43	1	1.86E-02
Homocysteine Degradation	9	1	1.86E-02
Ammonia Recycling	32	2	1.86E-02
Amino Sugar Metabolism	33	2	1.86E-02
Purine Metabolism	74	2	1.86E-02
Warburg Effect	58	2	1.86E-02
Tyrosine Metabolism	72	2	3.70E-02
Oxidation of Branched Chain Fatty Acids	26	2	3.70E-02
Phenylacetate Metabolism	9	1	3.70E-02
Phenylalanine and Tyrosine Metabolism	28	1	3.70E-02
Propanoate Metabolism	42	1	3.70E-02
Folate Metabolism	29	1	3.70E-02
Alanine Metabolism	17	1	3.70E-02
Glucose-Alanine Cycle	13	1	3.70E-02
Malate-Aspartate Shuttle	10	1	3.70E-02
Fatty acid Metabolism	43	1	4.45E-02
Beta Oxidation of Very Long Chain Fatty Acids	17	1	4.45E-02
Mitochondrial Beta-Oxidation of Short Chain Saturated Fatty Acids	27	1	4.45E-02
Mitochondrial Beta-Oxidation of Long Chain Saturated Fatty Acids	28	1	4.45E-02

Supplementary Table S5. Quantitative enrichment analysis in MetaboAnalyst 4.0 (colon tissue matrix). 35 identified metabolites discriminating (VIP-value > 1.0 and/or P-value < 0.05) between the non-transformed and the transformed state in the colon tissue matrix. FDR = false discovery rate.

**Affected pathways in the colon tissue matrix**	**Total compounds in pathway**	**Hits**	**FDR**
Glycerolipid Metabolism	25	1	7.28E-03
Riboflavin Metabolism	20	1	7.28E-03
Glycerol Phosphate Shuttle	11	1	7.28E-03
Pyrimidine Metabolism	59	4	1.36E-02
Arginine and Proline Metabolism	53	2	1.36E-02
Glutamate Metabolism	49	2	1.36E-02
Phenylalanine and Tyrosine Metabolism	28	1	1.36E-02
Propanoate Metabolism	42	1	1.36E-02
Valine, Leucine and Isoleucine Degradation	60	1	1.36E-02
Lysine Degradation	30	1	1.36E-02
Amino Sugar Metabolism	33	1	1.36E-02
Folate Metabolism	29	1	1.36E-02
Alanine Metabolism	17	1	1.36E-02
Glucose-Alanine Cycle	13	1	1.36E-02
Warburg Effect	58	1	1.36E-02
Ammonia Recycling	32	1	1.66E-02
Purine Metabolism	74	2	1.66E-02
Urea Cycle	29	2	1.66E-02
Aspartate Metabolism	35	1	1.66E-02
Malate-Aspartate Shuttle	10	1	1.66E-02
Glycine and Serine Metabolism	59	2	1.66E-02
Cysteine Metabolism	26	2	1.66E-02
Glutathione Metabolism	21	2	1.66E-02
Beta-Alanine Metabolism	34	3	1.89E-02
Pantothenate and CoA Biosynthesis	21	1	2.49E-02

Methionine Metabolism	43	1	2.49E-02
Homocysteine Degradation	9	1	2.49E-02
Tyrosine Metabolism	72	3	3.86E-02
Tryptophan Metabolism	60	2	4.07E-02
Oxidation of Branched Chain Fatty Acids	26	2	4.58E-02
Carnitine Synthesis	22	2	4.58E-02
Catecholamine Biosynthesis	20	1	4.69E-02
Phytanic Acid Peroxisomal Oxidation	26	1	4.69E-02
